# Supplementary material for: Total Phenolic Content, Biomass Composition, and Antioxidant Activity of Selected Marine Microalgal Species with Potential as Aquaculture Feed
Source: Antioxidants (Basel). 2022 Jul 4;11(7):1320. doi: 10.3390/antiox11071320 (PMC9311600; doi:10.3390/antiox11071320)
Supplement: Supplementary file 1 [file antioxidants-11-01320-s001.zip › Supplementary Figures.pdf]

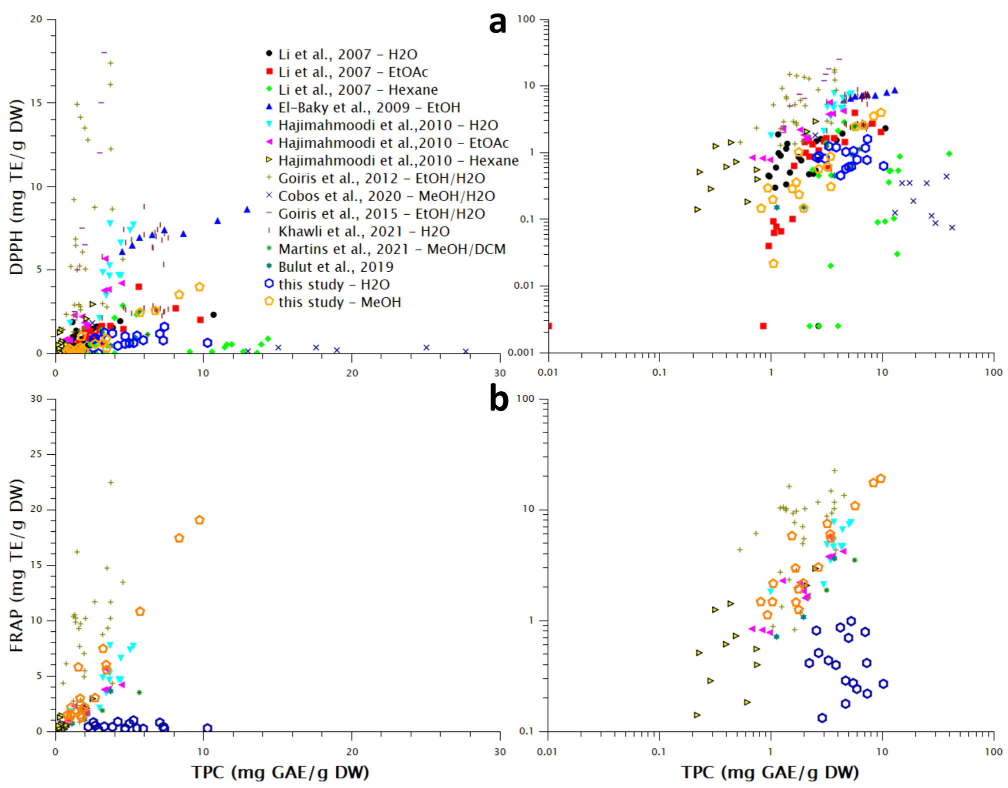

Figure S1. (a) Data of TPC vs. DPPH RSA and (b) TPC vs. FRAP from the current study and various references. On the right, the same data are plotted in log scale for clarity.

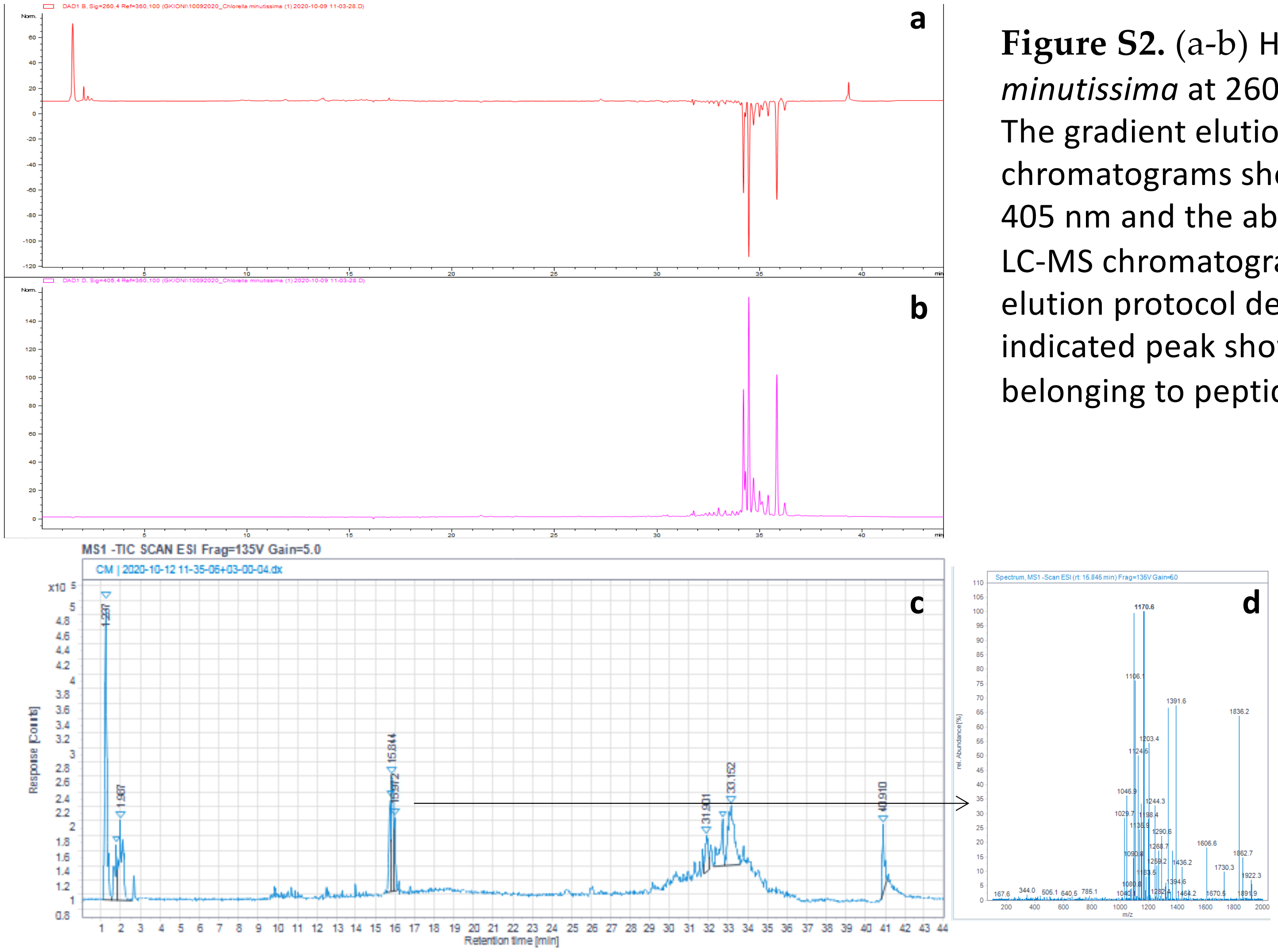

Figure S2. (a-b) HPLC analysis of 70 % methanol extract of *C. minutissima* at 260 nm (a) and 405 nm (b) on a C18 column. The gradient elution program is described in 2.8.1. The chromatograms show the presence of pigments detected at 405 nm and the absence of phenolics. (c) Negative ionization LC-MS chromatogram of the same extract with the same elution protocol described in 2.8.1. (d) Mass spectrum of indicated peak showing high molecular weight ions probably belonging to peptides which do not absorb at UV/vis.

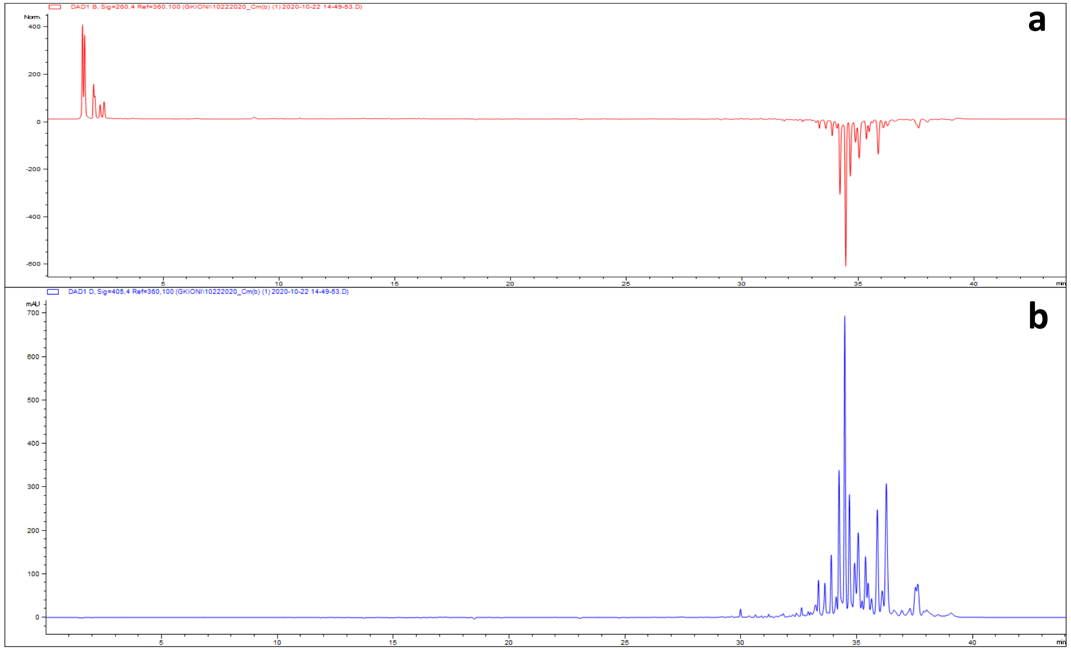

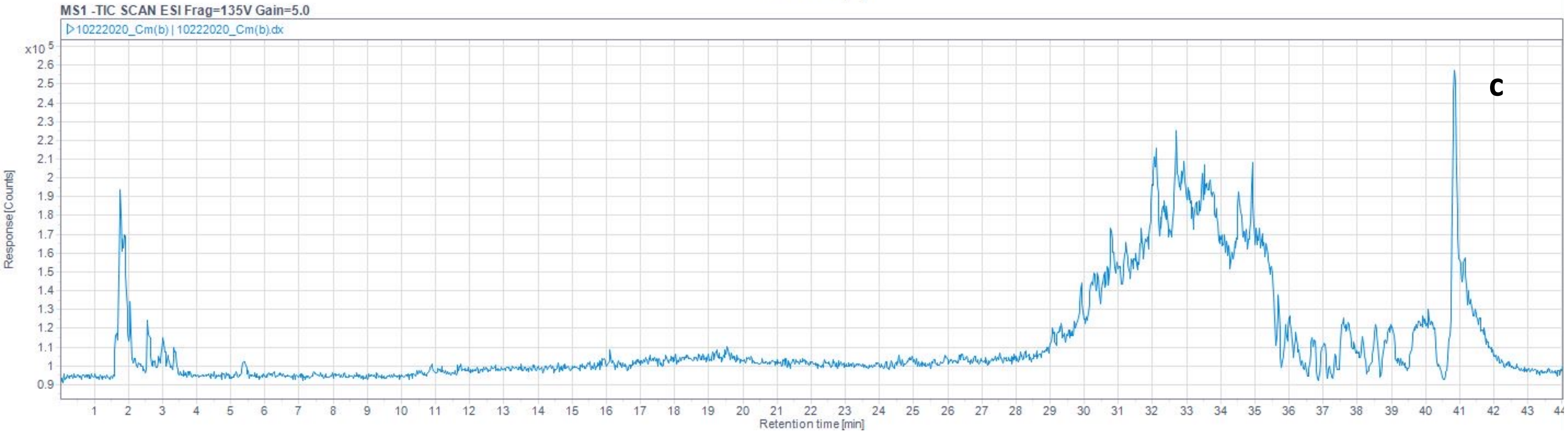

Figure S3. (a-b) HPLC analysis of 100 % methanol extract of *C. minutissima* obtained with protocol C at 260 nm (a) and 405 nm (b). The gradient elution program is described in 2.8.1. (c) Negative ionization LC-MS chromatogram of the same extract with the same elution protocol described in 2.8.1. The absence of phenolic compounds as well as the absence of peaks probably originating from peptides that were extracted with 70% methanol (Figure S2) are evident.

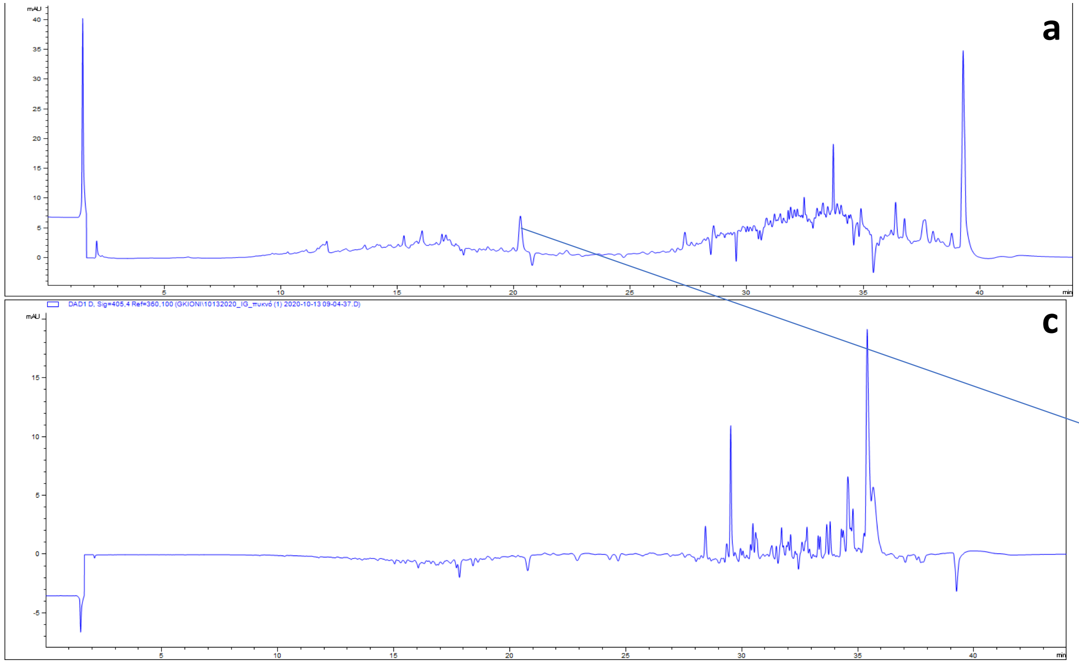

Figure S4. (a, c) HPLC analysis of 70 % methanol extract of *I. galbana* at 260 nm (a) and 405 nm (c). The gradient elution program is described in 2.8.1. (b) The absorption spectrum of indicated peak in the middle of the chromatogram shows the presence of an aromatic compound. (d) Negative ionization LC-MS chromatogram of the same extract with the same elution protocol described in 2.8.1.

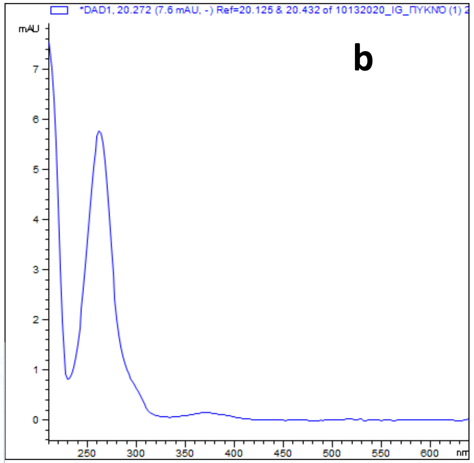

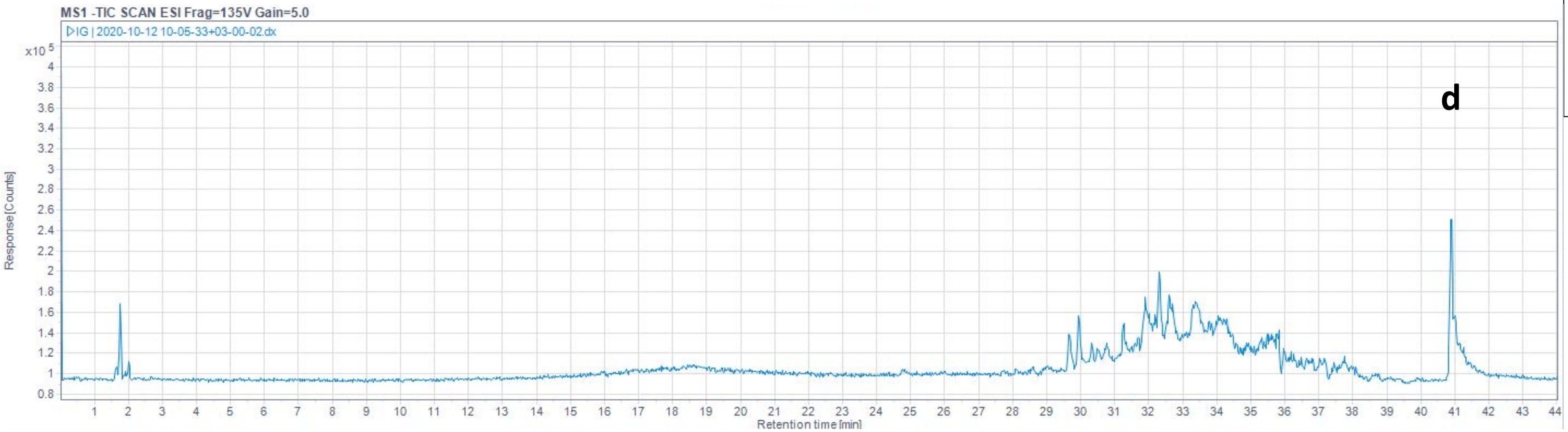

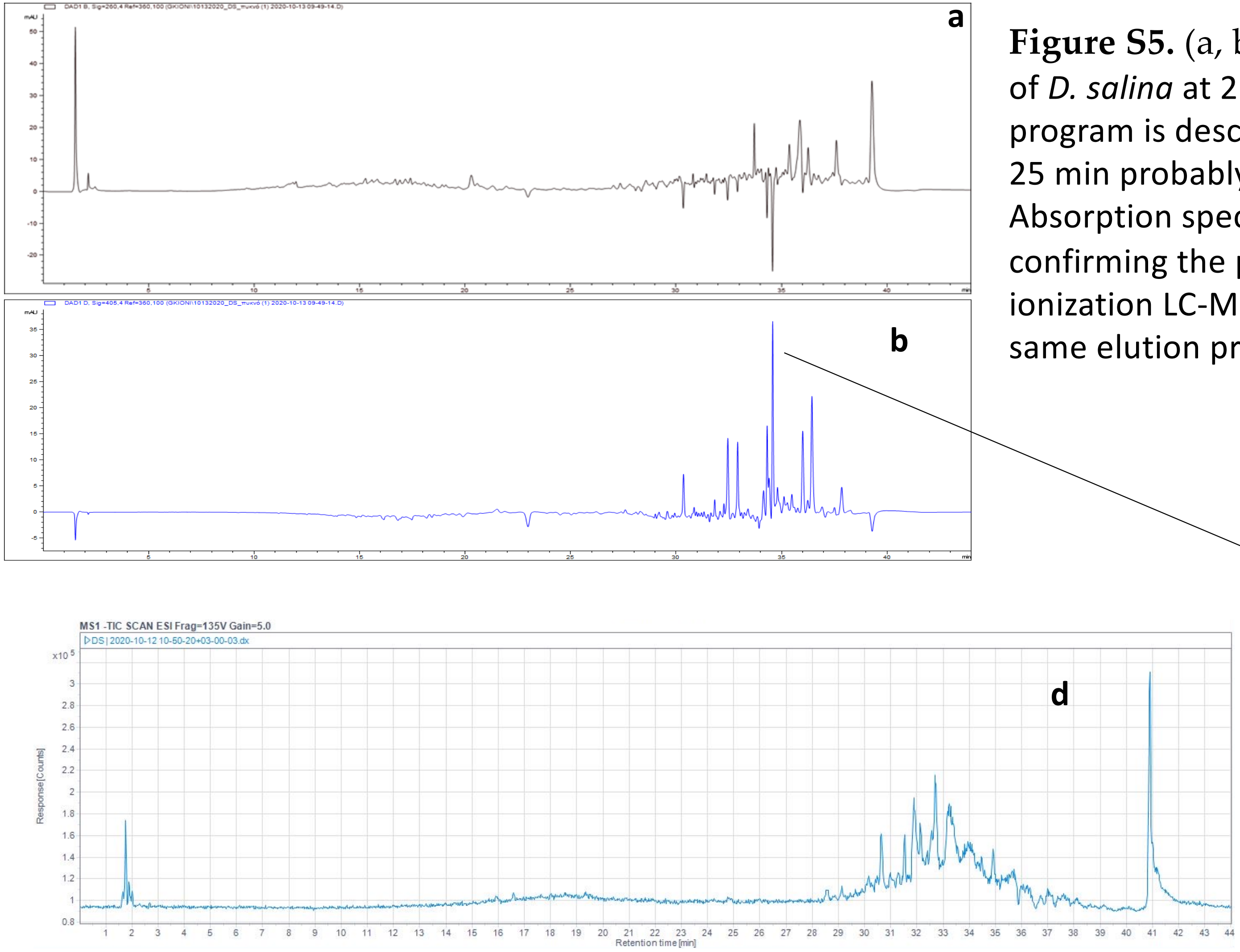

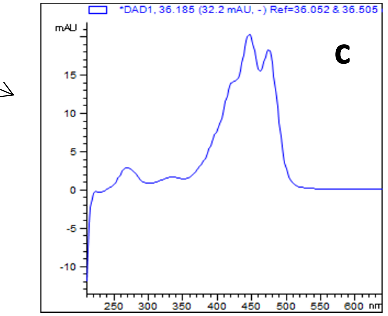

Figure S5. (a, b) HPLC analysis of 70 % methanol extract of *D. salina* at 260 nm (a) and 405 nm (b). The gradient elution program is described in 2.8.1. Very small peaks eluting at 10-25 min probably belong to aromatic compounds. (c) Absorption spectrum of the main peak in the pigment area confirming the presence of carotenoids. (d) Negative ionization LC-MS chromatogram of the same extract with the same elution protocol described in 2.8.1.

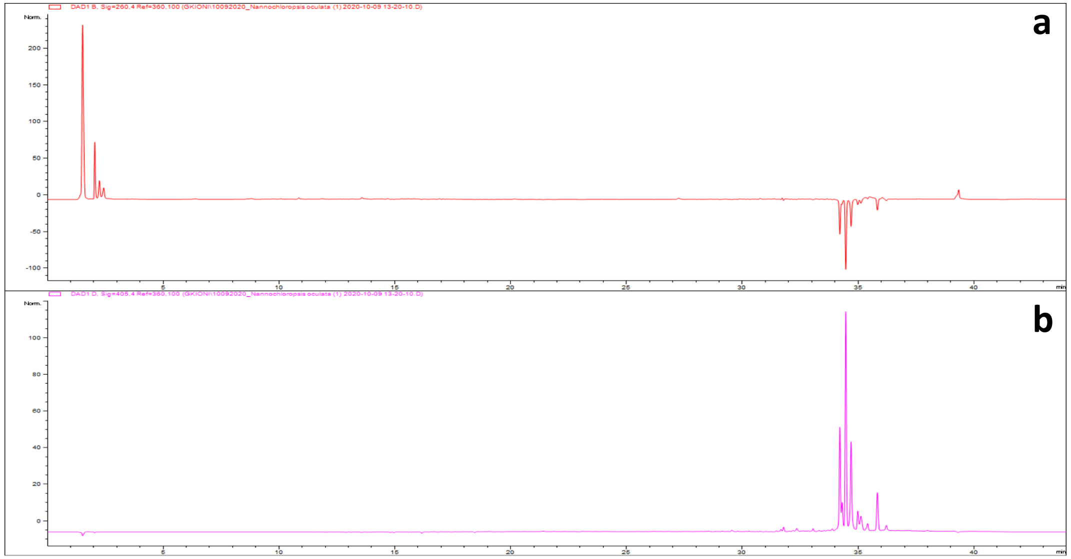

Figure S6. (a-b) HPLC analysis of the 70 % methanol extract of *N. oculata* at 260 nm (a) and 405 nm (b). The gradient elution program is described in 2.8.1. The chromatograms are dominated by pigments. (c) Negative ionization LC-MS chromatogram of the same extract with the same elution protocol described in 2.8.1. (d) Mass spectrum of peak at 10.5 min showing high molecular weight ions probably belonging to peptides which do not absorb at UV/vis.

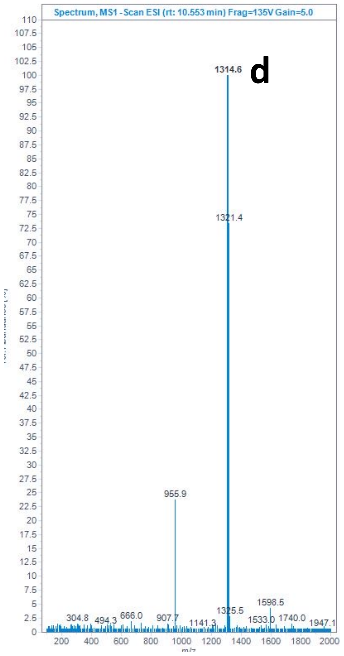

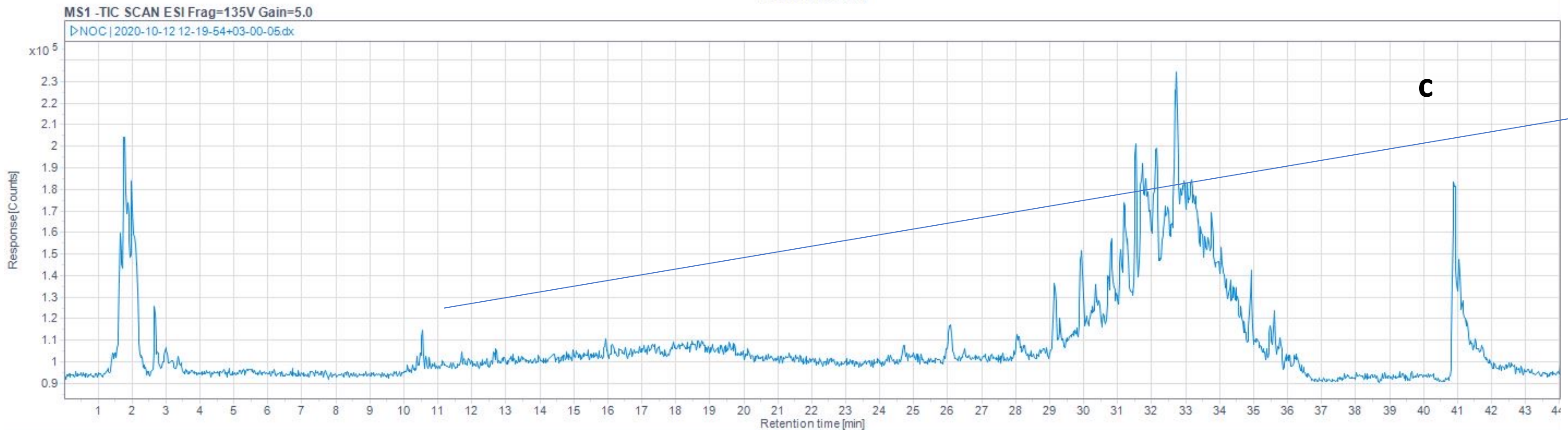

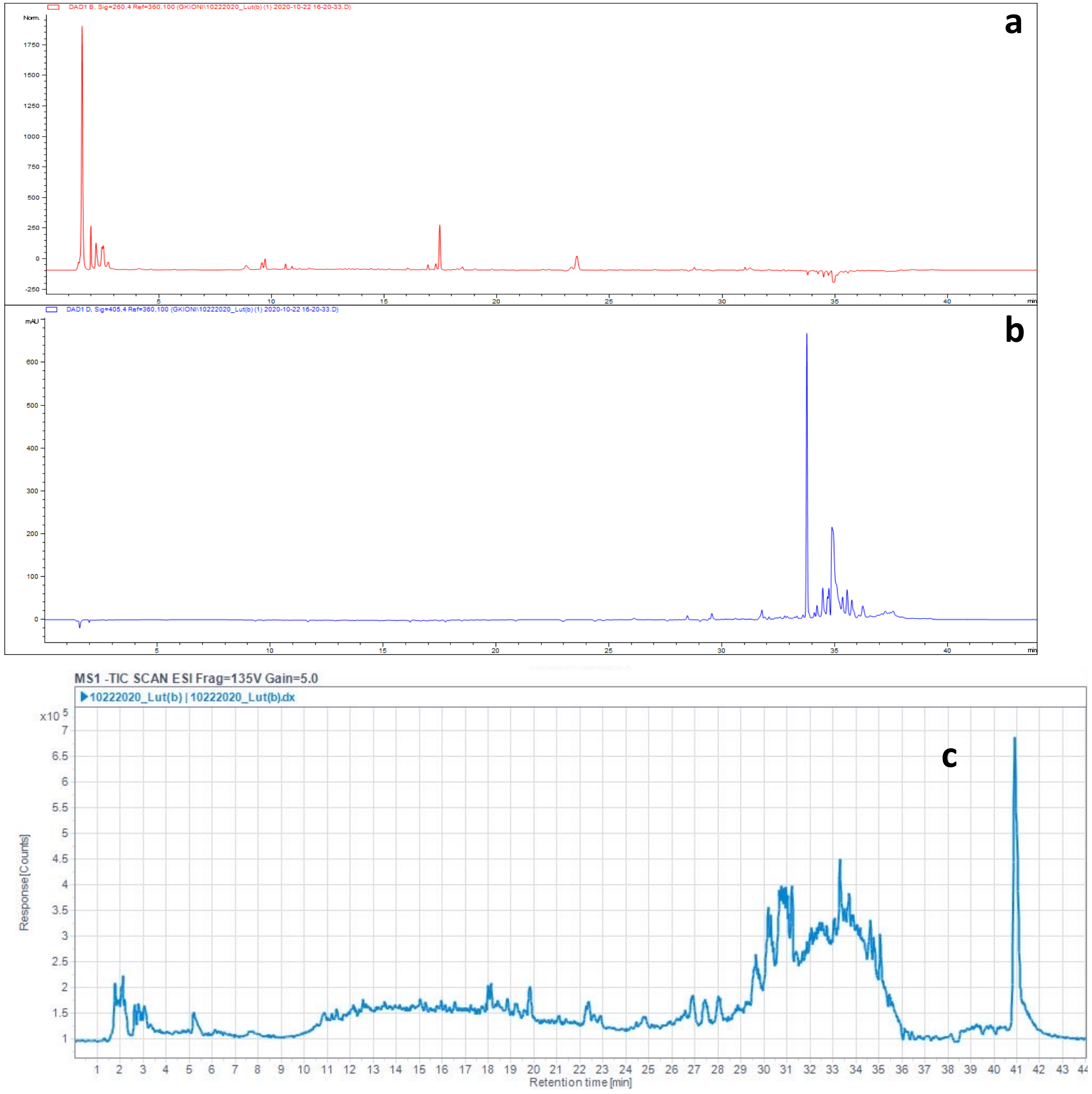

Figure S7. (a-b) HPLC analysis of 100 % methanol extract of *T. lutea* extracted with protocol C at 260 nm (a) and 405 nm (b). The gradient elution program is described in 2.8.1. (c) Negative ionization LC-MS chromatogram of the same extract with the same elution protocol described in 2.8.1. Apart from the presence of pigments, peaks of phenolic nature are present between 10 and 25 min in both (a) and (c).

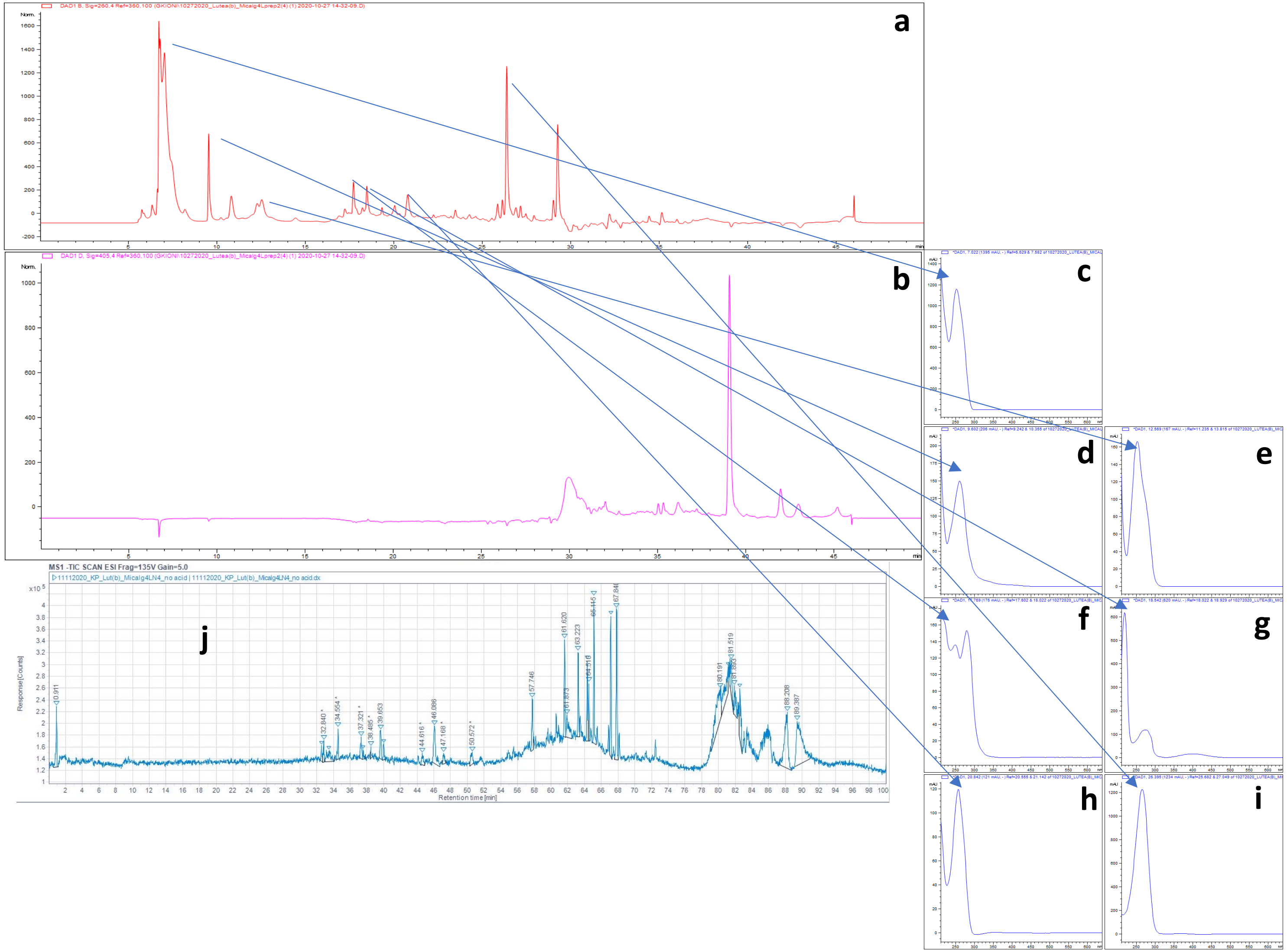

Figure S8. (a-b) Semipreparative HPLC analysis of 100 % methanol extract of *T. lutea* extracted with protocol C at 260 nm (a) and 405 nm (b). The gradient elution program is described in 2.8.3. (c-i) Absorption spectrum of indicated peaks demonstrating their aromatic nature. (j) Negative ionization LC-MS chromatogram of the same extract at the conditions described in 2.8.2. The examination of the mass spectra and of the literature did not allow us to identify any of the compounds. We analysed a set of standard phenolics (k), but they all eluted earlier than the first peaks of *T. lutea*.

k: LC-MS elution times of standard compounds

Elution Time (min)	Compound
4.28	Gallic acid
8.87	Protocatechuic acid
12.41	4-hydroxybenzoic acid
14.83	4-hydroxybenzaldehyde
15.64	Vanillic acid
16.18	(+)-Catechin
16.58	Caffeic acid
19.29	(-)-Epicatechin
20.12	p-Coumaric acid
21.01	(-)-Epigallocatechin 3-gallate
21.56	Salicylic acid
24.70	(-)-Epicatechin 3-gallate
29.62	Resveratrol
